# Factors associated with the quality of the diet of residents of a rural area in Southern Brazil

**DOI:** 10.11606/S1518-8787.2018052000267

**Published:** 2018-09-13

**Authors:** Mayra Pacheco Fernandes, Renata Moraes Bielemann, Anaclaudia Gastal Fassa

**Affiliations:** I Universidade Federal de Pelotas. Faculdade de Medicina. Departamento de Medicina Social. Programa de Pós-Graduação em Epidemiologia. Pelotas, RS, Brasil; II Universidade Federal de Pelotas. Faculdade de Medicina. Departamento de Medicina Social. Pelotas, RS, Brasil

**Keywords:** Adult, Aged, Food Consumption, Feeding Behavior, Socioeconomic Factors, Rural Population, Adulto, Idoso, Consumo de Alimentos, Comportamento Alimentar, Fatores Socioeconômicos, População Rural

## Abstract

**OBJECTIVE:**

To identify factors associated with a better quality of the diet of residents of a rural area in Southern Brazil.

**METHODS:**

This is a population-based, cross-sectional study with individuals aged 18 years or over living in the rural area of Pelotas, State of Rio Grande do Sul, Brazil. Food consumption was evaluated by a food frequency questionnaire of thirteen items, related to the consumption in the last week. We evaluated quality of the diet using the Adult Diet Quality Index (IQD-A). Healthy food received increasing scores while unhealthy food received decreasing scores, according to consumption frequency, amounting to scores from zero to 30. The total score was divided into tertiles. Individuals of the third tertile were classified with better quality of the diet. We investigated the association between quality of the diet and independent variables using multinomial logistic regression.

**RESULTS:**

We interviewed 1,519 individuals with mean IQD-A of 17.1 points (SD = 3.3) and a median of 17.0 (range of 10 to 25 points). Although the population studied kept the consumption of staple foods, the intake of industrialized food such as soft drinks, artificial juices, and unhealthy foods such as sweets was high. Older individuals presented seven times (95%CI 4.20–12.48) more chance of having a better quality of the diet. Women, individuals with higher economic status, those who worked in the sale of animals, or those who had diabetes were approximately twice as likely to be in the group with the best quality of the diet. Individuals whose families worked with fishing presented a 70% lower chance of being in the group of better quality of the diet.

**CONCLUSIONS:**

We identified that men, younger adults, individuals of lower socioeconomic level, and fishing families were in the group of higher vulnerability for the consumption of a diet with worse quality. Thus, public policies, especially educational policies, are needed to promote healthy eating in this group.

## RESUMO

**OBJETIVO:**

Identificar fatores associados à melhor qualidade da dieta de moradores da zona rural do Sul do Brasil.

**MÉTODOS:**

Foi realizado estudo transversal de base populacional com moradores de 18 anos ou mais, da zona rural de Pelotas, RS, Brasil. O consumo alimentar foi avaliado por um questionário de frequência alimentar de 13 itens, referente ao consumo da última semana. A qualidade da dieta foi avaliada pelo Índice de Qualidade da Dieta de Adultos (IQD-A). Os alimentos saudáveis receberam pontuação de forma crescente enquanto os alimentos não saudáveis receberam pontuação decrescente conforme a frequência de consumo, formando escores de zero a 30. A pontuação total foi dividida em tercis. Indivíduos do 3º tercil foram classificados com melhor qualidade da dieta. A associação entre a qualidade da dieta e as variáveis independentes foi investigada usando-se regressão logística multinomial.

**RESULTADOS:**

Foram entrevistados 1.519 indivíduos com média de IQD-A de 17,1 pontos (DP = 3,3) e mediana de 17,0 (amplitude de 10 a 25 pontos). Embora a população estudada mantenha o consumo de alimentos básicos, a ingestão de alimentos industrializados como refrigerantes, sucos artificiais e de alimentos não saudáveis como doces foi alta. Indivíduos mais velhos apresentaram sete vezes (IC95% 4,20–12,48) mais chance de ter melhor qualidade da dieta. As mulheres, indivíduos com maior nível econômico, que trabalhavam com venda de animais ou que possuíam diabetes apresentaram cerca de duas vezes mais chance de estarem no grupo de melhor qualidade da dieta. Indivíduos cujas famílias trabalhavam com pesca apresentaram 70% menor chance de estar no grupo de melhor qualidade da dieta.

**CONCLUSÕES:**

Identificou-se um grupo de maior vulnerabilidade ao consumo de dieta com pior qualidade, sendo esse constituído por homens, adultos mais jovens, indivíduos de menor nível econômico e de família de pescadores. Assim, são necessárias políticas públicas, especialmente educacionais, com o fim de promover uma alimentação saudável para esse grupo.

## INTRODUCTION

Rural areas in Brazil are undergoing a process of population aging^1^, as well as an increase in the prevalence of overweight^2^ and chronic non-communicable diseases (NCD)^3^. Inadequate diet is an important risk factor for NCD, and it is also associated with disability and premature death^4^. A systematic review has shown that the Brazilian population needs to improve the quality of the diet, given the low consumption of fruits, vegetables, milk, and dairy products and the high consumption of fat^5^.

The evaluation of the quality of the diet in epidemiological studies is a challenge. Among the most accurate dietary methods to evaluate dietary consumption or eating habits, we have the 24-hour recall or food record; however, it is complex to be used in population studies. In this sense, some studies have used the Diet Quality Index (IQD). The IQD is constructed from food intake information from the application of a food frequency questionnaire (FFQ), which, in turn, can consist of a list of food^6^ or food consumption markers^7^.

The rural area, especially in areas focused on family agriculture, used to be characterized by the production of food for subsistence^8^. However, this scenario has been changing with the expansion of monoculture^9^. This aspect, in addition to the barriers to the access to consumer goods (such as low income and low education level), the great distances and difficulty of access to public transportation, stores, and health services^10^ may be modifying the quality of the diet of this population.

There are few studies on the quality of the diet in rural areas. According to the Brazilian Household Budget Survey (POF), rural areas presented higher consumption of traditional food, while urban areas presented higher consumption of ready-to-eat or processed food^11^. In the National Health Survey (PNS)^3^, rural areas had higher consumption of beans and fat, as well as lower consumption of fruits, vegetables, sweets, and soft drinks compared to urban areas. Studies on the quality of the diet in the urban area show that women and older persons^6^ ^,^ ^12^ with higher education level^6^ ^,^ ^12^ and higher socioeconomic level^12^ present a better quality of diet. However, there are no studies on factors associated with the quality of diet in rural areas in Brazil.

Considering the importance of an adequate diet in health promotion, as well as the lack of information on the factors associated with the quality of diet in rural areas, this study aimed to identify the socioeconomic and demographic factors and the comorbidities associated with the best quality of diet of residents of the rural area.

## METHODS

Between January and June 2016, we carried out a population-based, cross-sectional study with residents of the rural area of Pelotas, State of Rio Grande do Sul, Brazil, aged 18 years or over. This study is part of a research consortium that has aimed to know the health characteristics of this population. We excluded adults or older adults with enteral or parenteral nutrition, institutionalized individuals, persons with mental disabilities that made them unable to answer the questionnaire, and individuals who were bedridden and who could not communicate or whose caregiver or guardian could not report food consumption.

To estimate the prevalence of food consumption, we used as reference the prevalence of 10.8% for chicken consumption^3^. Thus, with a 95% confidence level, an acceptable error of 2.5 percentage points, a delineation effect of 2.0, and 10% for losses and refusals, the required sample was 1,302 individuals.

The sample was selected in two stages, and the census tracts defined by the Brazilian Institute of Geography and Statistics (IBGE) were the primary sample unit. The 50 rural census tracts were listed according to the 2010 Census^1^. Tracts with seven or fewer households were excluded. This resulted in 45 census tracts, of which 24 were systematically selected. In each tract, 30 residences were selected, which resulted in 720 households.

For the stage of recognition of the households, we used the Google Earth software along with a virtual map of the state of Rio Grande do Sul, provided by IBGE. From the aerial images, we determined centers with the largest household cluster, in each tract, ordered in a decreasing way in relation to the number of houses identified by the satellite images. To select the sampled households, we randomly selected the direction to be traveled in the first center (with the largest household cluster), until we selected 30 houses. When we reached the end of the direction indicated without identifying 30 houses, we restarted the process going to the next road, to the right of the first one. After tracing the entire first center, if we did not reach a total of 30 houses, we would go to the second center, repeating the process previously described. The methodological article of this study, available in this same edition, presents more details on the sampling^13^.

### Dependent Variables

The quality of the diet was evaluated by the methodology proposed by Gomes et al.^7^, who have carried out a study with older adults from the urban area of the same city. The adapted version of the FFQ consisted of 13 markers of food consumption, related to last week’s consumption of vegetables, fruits, beans, milk, whole foods, red meat, chicken, fried food, preserved or canned food, cured food, frozen and ready-to-eat food (processed food), sweets, soft drinks, or artificial juices. The consumption of fast food was not questioned.

Food consumption was grouped into four categories: did not eat, 1–3 days, 4–6 days, and daily. We assigned points according to consumption categories and type of food, ranging from zero to three^7^. Thus, healthy foods receive an increasing score (no consumption = zero points, consumption every day = three points), while unhealthy foods received a decreasing score (no consumption = three points, consumption every day = zero points) (Box).

**Box:** Description of the score used to calculate the Adult Diet Quality Index (IQD-A).

Components of the IQD-A	Score by category of consumption
Healthy^a^ – fruits, vegetables, beans, milk, meat or chicken, whole food	0 – Did not eat in the last week
	1 – Ate 1–3 days/week
	2 – Ate 4–6 days/week
	3 – Ate everyday
Unhealthy^b^ – fried food, cured and/or preserved food, frozen food (industrialized), soft drinks, artificial juices, sweets	3 – Did not eat in the last week
	2 – Ate 1–3 days/week
	1 – Ate 4–6 days/week
	0 – Ate everyday

Source: adapted from Gomes et al.^7^ (2016).

^a^ Higher mean scores indicate higher frequency of consumption.

^b^ Higher mean scores indicate lower frequency of consumption.

The sums of the points amounted to a total score from zero to 30, resulting in the Adult Diet Quality Index (IQD-A). The total score was divided into tertiles, and individuals in the 3rd tertile were considered as having the highest score. The highest score indicates a better quality of the diet, that is, a higher frequency of consumption of healthy food and a lower frequency of consumption of unhealthy food.

### Independent Variables

We characterized the sample in the socioeconomic, demographic, occupational, and health aspects: sex (observed), race (observed and subsequently dichotomized into white and non-white), age (complete years, later classified into 18 to 24 years, 25 to 39 years, 40 to 59 years, and 60 years or over), education level (in full years, subsequently categorized into zero to four years, five to eight years, and nine years or more), marital status (with partner or without partner), socioeconomic level, collected according to an instrument proposed by the Brazilian Association of Research Companies (ABEP)^14^, which considers the ownership of goods (television, radio, car, washing machine, VCR or DVD, refrigerator, and freezer), as well as education level of the household head, number of toilets/bathrooms in the household, and presence of a monthly cleaning worker, which was later categorized into A or B (highest), C, and D or E (lowest), and number of residents (categorized into 1, 2, 3, and 4 or more). We also evaluated the rural activities carried out by the interviewee or by a member of the family, such as work with fishing, selling of animals, and selling of agricultural products, with dichotomous answers (no, yes). The morbidities of arterial hypertension and diabetes mellitus were obtained from the self-report of medical diagnosis of each of the diseases. To test the agreement, we used the question “Do you know how to read and write?”, obtained from a reduced quality control questionnaire, applied to 10% of respondents. We obtained a coefficient of 0.76 using the Kappa test. We could not use questions about food consumption, as the answers could vary over time.

### Data Analysis

The data were collected in tablets and inserted directly into the electronic database by the questionnaire applied in RedCap (Research Electronic Data Capture). The analyses were carried out in the program SPSS, version 12.1. (Stata Corp, College Station, United States). We used the descriptive analysis to characterize the sample. In order to evaluate which food groups or combinations contributed to the category of better quality of the diet, we calculated the mean score of each group according to the tertiles of quality of the diet. We estimated the measures of effect using multinomial logistic regression to obtain the crude and adjusted odds ratio according to IQD-A categories, with low quality of diet as the reference category. The adjusted analysis considered two hierarchical levels. At the first level, we included the demographic and socioeconomic variables (sex, age, race, marital status, education level, number of residents, and work with fishing, sale of animals, and sale of agricultural products) which presented p < 0.20 in the crude analysis. At the second level, we added hypertension and diabetes mellitus. Variables with p < 0.20 were kept in the final model. Statistical associations with p ≤ 0.05 were considered significant. We considered the effect of the sampling design in all analyses, using the *svy* command. We also used weighting considering the number of fixed households according to IBGE data and those sampled within the districts of the rural area of Pelotas.

### Ethical Aspects

The project was approved by the Research Ethics Committee of the Faculdade de Medicina of the Universidade Federal de Pelotas (Protocol 51399615.7.0000.5317). The individuals of the research or their guardians signed the informed consent.

## RESULTS

We sampled 1,697 individuals eligible for the study. Losses and refusals amounted to 178 persons (10.5%), and most of them were males (70.8%) and aged between 40 and 59 years (7.6%). Thus, we interviewed 1,519 adults. All participants answered the FFQ.

Most of the sample consisted of women (51.7%), individuals aged 40 years or over (66.0%), white (85.1%), and with no partner (60.3%). Regarding the socioeconomic aspects, 75.6% of the participants had less than eight years of study and a fourth belonged to the class D or E; additionally, almost half of the households had four or more residents. Regarding the economic activities carried out by the interviewee or by a family member, one third sold agricultural products, 11% sold animals, and 4.9% worked with fishing (Table 1).

**Table 1:** Description of the sample according to the demographic, socioeconomic, and health variables. Pelotas, State of Rio Grande do Sul, Brazil, 2016. (n = 1,519)

Variable	n	% (95%CI)
Sex		
Male	734	48.3 (46.2–50.3)
Female	785	51.7 (49.7–53.7)
Age (years)		
18–24	174	11.4 (10.0–12.9)
25–39	341	22.6 (18.9–26.1)
40–59	593	39.2 (36.8–41.6)
60 or over	411	26.8 (23.4–30.2)
Race		
White	1,296	85.1 (79.6–90.6)
Non-white	223	14.9 (9.4–20.4)
Marital status		
Without partner	920	60.3 (55.9–64.6)
With partner	599	39.7 (35.4–44.1)
Education level (full years)		
0–4	582	38.7 (32.2–45.1)
5–8	558	36.9 (32.6–41.2)
9 or more	369	24.4 (18.0–30.9)
Number of residents		
1	94	6.2 (4.5–7.9)
2	388	25.6 (20.2–31.1)
3	395	26.4 (22.1–30.6)
4 or more	635	41.8 (33.7–49.9)
Socioeconomic level (ABEP)		
A or B	301	20.0 (14.3–25.7)
C	814	53.7 (48.2–59.3)
D or E	388	26.3 (20.1–32.4)
Work with fishing		
No	1,448	95.1 (88.4–101.7)
Yes	64	4.9 (-1.7–11.6)
Selling of animals		
No	1,341	89.0 (84.4–93.6)
Yes	171	11.0 (6.4–15.6)
Selling of agricultural products		
No	1,010	67.3 (52.7–82.0)
Yes	502	32.7 (18.0–47.3)
SAH		
No	994	65.7 (62.2–69.2)
Yes	522	34.3 (30.8–37.8)
DM		
No	1,363	89.8 (88.3–91.3)
Yes	153	10.2 (8.7–11.7)

ABEP: *Associação Brasileira de Empresas de Pesquisa* (Brazilian Association of Research Companies); SAH: systemic arterial hypertension; DM: diabetes mellitus

Mean IQD-A score was 17.1 (SD = 3.3) and median was 17.0, with a range between 10 and 25 points. We show the mean points of each component (food group or combination) of the IQD-A according to the tertiles of quality of the diet in the Figure. In the tertile of better quality of the diet, we found the highest means for healthy food, which indicates higher consumption, and for unhealthy food, which indicates lower consumption. However, the mean score of the consumption of meat or poultry and whole foods is less than two, which indicates a lower frequency of consumption in relation to other healthy foods. The mean score for the consumption of soft drinks, artificial juices, or sweet is also less than two, which indicates a higher frequency of consumption in relation other unhealthy foods in this tertile. In all the tertiles of quality of the diet, we observed a low frequency in the consumption of frozen or industrialized food, as this component of the IQD-A was close to three, which refers to the maximum score attributed according to the frequency of consumption (Box).

**Figure:**
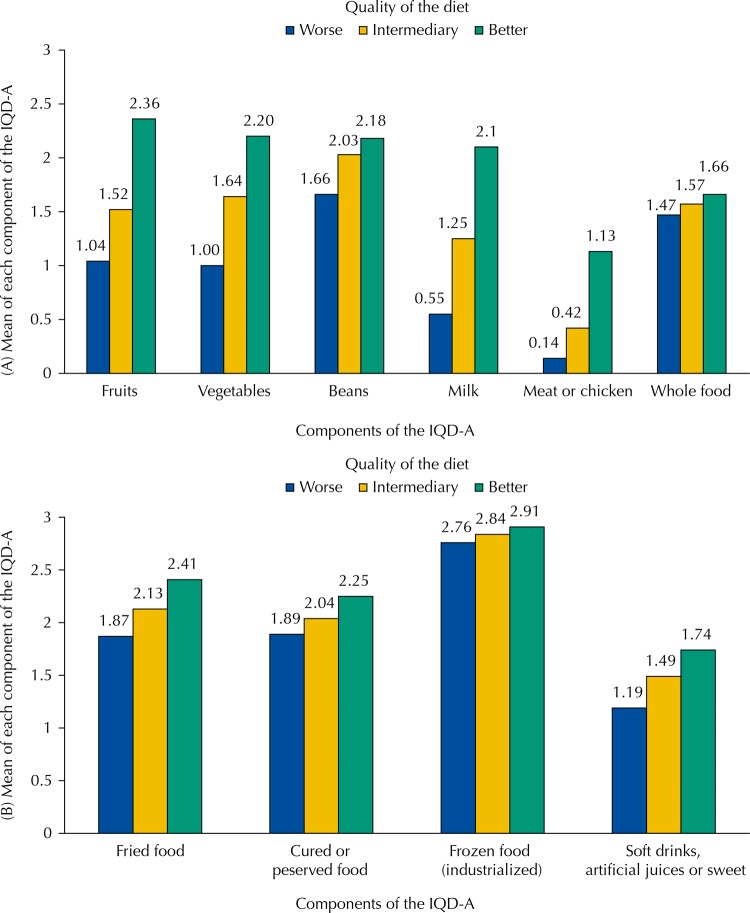
Mean of each component according to the categories of the IQD-A. Pelotas, State of Rio Grande do Sul, Brazil, 2016. (n = 1,519)

IQD-A: Adult Diet Quality Index

(A) Higher means indicate higher frequency of consumption.

(B) Higher means indicate lower frequency of consumption.

In the crude analysis, sex, age, marital status, number of residents, socioeconomic level, work with fishing, and diagnosis of hypertension or diabetes were significantly associated with high quality of diet (Table 2). After the adjusted analysis (Table 3), marital status, number of residents, and medical diagnosis of hypertension lost significance, and the sale of animals was associated with the outcome. Women were 2.4 times more likely (95%CI 1.61–3.44) to have better quality of diet than men. Age is directly associated with better quality of diet; individuals aged 60 years or over were seven times more likely (95%CI 4.20–12.48) to be in the tertile of better diet than those aged 18 to 24 years. Individuals in class A or B were 2.4 times more likely (95%CI 1.49–3.76) to have a better quality of diet than individuals in class D or E.

**Table 2:** Crude analysis between quality of the diet and demographic, economic, and health variables. Pelotas, State of Rio Grande do Sul, Brazil, 2016. (n = 1,519)

Variable	Quality of the diet^a^			

	Intermediary		Better	
	
	OR (crude)	p^c^	OR (crude)	p^c^
Sex		0.142		< 0.001
Male	1.00		1.00	
Female	1.21 (0.93–1.59)		2.23 (1.62–3.07)	
Age (years)		< 0.001^d^		< 0.001^d^
18–24	1.00		1.00	
25–39	1.54 (1.00–2.39)		1.78 (1.10–2.90)	
40–59	2.24 (1.54–3.26)		2.79 (1.85–4.19)	
60 or over	2.87 (1.97–4.17)		7.49 (4.51–12.44)	
Race		0.320		0.126
White	1.00		1.00	
Non-white	0.82 (0.55–1.23)		0.74 (0.50–1.09)	
Marital status		0.011		0.047
Without partner	1.00		1.00	
With partner	0.70 (0.54–0.92)		0.77 (0.59–0.99)	
Education level (full years)		0,161		0,161
0–4	1.00		1.00	
5–8	0.72 (0.52–0.99)		0.67 (0.49–0.92)	
9 or more	0.80 (0.58–1.09)		0.93 (0.68–1.29)	
Number of residents		0.698^d^		0.001^d^
1	1.22 (0.91–1.64)		2.67 (1.51–4.72)	
2	1.08 (0.74–1.57)		2.10 (1.33–3.30)	
3	1.01 (0.51–2.00)		1.71 (1.19–2.48)	
4 or more	1.00		1.00	
Socioeconomic level (ABEP)^b^		0.545^d^		0.049^d^
A or B	1.18 (0.71–1.94)		1.83 (0.99–3.4)	
C	1.05 (0.67–1.65)		1.08 (0.74–1.58)	
D or E	1.00		1.00	
Work with fishing		0.001		0.018
No	1.00		1.00	
Yes	0.48 (0.32–0.71)		0.27 (0.09–0.78)	
Selling of animals		0.158		0.053
No	1.00		1.00	
Yes	1.33 (0.88–2.01)		1.55 (0.99–2.41)	
Selling of agricultural products		0.552		0.098
No	1.00		1.00	
Yes	1.11 (0.78–1.58)		0.61 (0.34–1.10)	
SAH		0.133		< 0.001
No	1.00		1.00	
Yes	1.21 (0.94–1.56)		1.91 (0.54–1.04)	
DM		0.038		< 0.001
No	1.00		1.00	
Yes	1.78 (1.03–3.07)		3.34 (1.96–5.69)	

ABEP: *Associação Brasileira de Empresas de Pesquisa* (Brazilian Association of Research Companies); SAH: systemic arterial hypertension; DM: diabetes mellitus

^a^ Reference category: worst quality of diet.

^b^ Highest number of missing data: 32.

^c^ Multinomial logistic regression.

^d^ Linear trend.

**Table 3:** Factors associated with better quality of the diet among adults living in rural areas. Pelotas, State of Rio Grande do Sul, Brazil, 2016. (n = 1,519)

Variable	Quality of the diet^a^			

	Intermediary		Better	
	
	OR (adjusted)	p^b^	OR (adjusted)	p^b^
Sex		0.129		< 0.001
Male	1.00		1.00	
Female	1.25 (0.93–1.69)		2.35 (1.61–3.44)	
Age (years)		< 0.001^c^		< 0.001^c^
18–24	1.00		1.00	
25–39	1.57 (1.02–2.42)		1.82 (1.18–2.80)	
40–59	2.37 (1.64–3.42)		2.86 (1.86–4.39)	
60 or over	3.19 (2.12–4.80)		7.23 (4.20–12.48)	
Socioeconomic level (ABEP)		0.769^c^		0.001^c^
A or B	1.08 (0.67–1.76)		2.37 (1.49–3.76)	
C	0.97 (0.60–1.57)		1.34 (1.01–1.78)	
D or E	1.00		1.00	
Number of residents		0.056		0.056
1	0.83 (0.39–1.74)		1.78 (1.22–2.58)	
2	0.85 (0.55–1.31)		1.40 (0.87–2.26)	
3	1.25 (0.92–1.70)		1.90 (1.01–3.59)	
4 or more	1.00		1.00	
Work with fishing		< 0.001		0.005
No	1.00		1.00	
Yes	0.48 (0.33–0.70)		0.30 (0.14–0.67)	
Selling of animals		0.193		0.020
No	1.00		1.00	
Yes	1.33 (0.86–2.05)		1.84 (1.11–3.06)	
Selling of agricultural products		0.937		0.051
No	1.00		1.00	
Yes	0.99 (0.67–1.44)		0.57 (0.32–1.00)	
DM		0.199		0.003
No	1.00		1.00	
Yes	1.41 (0.82–2.40)		2.13 (1.33–3.42)	

ABEP: *Associação Brasileira de Empresas de Pesquisa* (Brazilian Association of Research Companies); DM: diabetes mellitus

^a^ Reference category: worst quality of diet.

^b^ Multinomial logistic regression.

^c^ Linear trend.

Regarding the variables of rural activity, individuals who had family members working with fishing had a 70% lower chance of being in the category of better quality of diet (p = 0.005). However, individuals who had family members working with the sale of animals had an 84% greater chance of being in the category of better quality of diet (p = 0.020). Individuals who reported a medical diagnosis of diabetes mellitus were twice as likely (95%CI 1.33–3.42) to have a better quality of diet.

## DISCUSSION

Regarding the quality of the diet, almost 50% of the adults and older adults in the rural area of Pelotas reached only half of the maximum score. Among the evaluated foods, those that contributed the least to the tertile of better quality of the diet were meat or chicken and whole foods, as they had a lower frequency of consumption than other healthy foods, and soft drinks, artificial juices, or sweet, as they had a higher frequency of consumption than other unhealthy foods. Women, individuals with diabetes, older persons, those with a higher socioeconomic level, and those who worked or had relatives who worked in the sale of animals were more likely to be in the tertile of better quality of the diet. On the other hand, individuals whose families worked with fishing presented a lower chance of having a better quality of the diet.

The highest concentration of women and older individuals in the group of better quality of the diet is consistent with the studies carried out in the urban area^6^ ^,^ ^12^, while the association with the socioeconomic level is controversial^6^ ^,^ ^12^. Women generally have better nutrition, health and body care, greater nutritional knowledge, and include healthier foods in the diet when compared to men^15^ ^,^ ^16^. The better quality of the diet of older individuals may be related to the formation of eating habits at a time when there was a low supply of processed or ultra-processed foods^17^. The increase in the prevalence of chronic diseases in older adults increases the search for health services, which boosts the opportunity to receive guidelines that contribute to better food choices^18^ and makes adherence to a healthy lifestyle necessary for survival. We also have survival bias in older adults, since older individuals with poorer quality of the diet have a higher risk of death and thus, survivors can concentrate individuals with better quality of the diet.

Individuals with higher purchasing power have greater access to healthier foods^19^, which may explain the higher proportion of persons in the higher socioeconomic classes in the tertile of better quality of diet. Expenditure with food is the second most important in family expenses and the one with the greatest weight in the budget of rural families and low income families^20^. However, this relation has not been fully elucidated, as studies show a positive effect of the economic level on the quality of the diet with increased consumption of fruits or vegetables^17^ ^,^ ^21^, but they also show a high consumption of food rich in saturated fat and simple carbohydrates. Thus, income can influence food consumption, without it being necessarily reflected in the quality of food^21^.

Several studies have found an association between education level and the quality of the diet^6^ ^,^ ^12^ ^,^ ^22^. In this study, education level was correlated at the economic level and, therefore, we excluded it from the final model. Individuals with higher education have more information on how and why to have a healthy diet^12^. Similarly to other studies conducted in the urban area, race and marital status were not associated with quality of the diet^6^ ^,^ ^12^.

As for rural activities, the higher proportion of individuals who have family members working with fishing in the group of poorer quality of the diet and the higher proportion of individuals who have family members working with the sale of animals in the tertile of better quality of the diet may be related to the residual confusion of the socioeconomic level. The organization of routes and access to services and the quality of the diet in the rural district of Pelotas, where the main economic activity is fishing, is similar to the peripheral and less developed urban area. In addition, work with fishing requires long working hours, which may hinder the access to healthy food. On the other hand, the sale of animals could be related to a greater diversification of economic activity.

Consistent with the literature^23^, a greater proportion of individuals with diabetes were in the group of better quality of the diet, but this association is affected by reverse causality, as, after the diagnosis of the disease, individuals are guided to reduce the consumption of simple carbohydrates^24^. However, individuals diagnosed with hypertension did not present better quality of diet. This could be related to a greater emphasis on salt reduction than on reduced fat consumption^25^, or lower adherence of individuals with hypertension to this treatment aspect.

This is the first population-based study on the quality of the diet of individuals living in the rural area. The low percentage of losses and refusals reinforces the representativeness of the sample studied. However, the study presents limitations related to the measurement of the outcome. The higher score of the index of quality of diet indicates a higher frequency of consumption of healthy food and a lower frequency of consumption of unhealthy food. However, there is no validation of the method that allows us assigning cutoff points to the IQD-A to characterize the diet as of high or low quality. In order to increase the comparability with the study by Gomes et al.^7^, we chose not to consider the fat content in milk and meat, which may cause misclassification of the subjects, overestimating the tertile of better quality of diet. We also highlight that because of the grouping of some foods for the score, we lost the specific information of each food. The FFQ is an instrument that can be easily applied and understood by the interviewees; however, the one-week recall, if on the one hand minimizes memory bias, on the other hand does not reflect the long-term food habit, including seasonal variabilities. Another limitation is related to potential inaccuracies in the classification of the socioeconomic level according to the ABEP criterion^14^, as this classification was developed for the urban area.

We conclude that public policies are needed to promote the low consumption of industrialized food and encourage the decreased consumption of soft drinks, artificial juices, or sweets and increased consumption of fruits and vegetables. This can be inserted in the context of policies for crop diversification, including cultivation for own consumption. These policies should prioritize low-income families.

The nutritional transition seems more advanced in younger adults. Considering that eating habits are formed in childhood and the role of children in transforming family habits, it is important to include food and nutrition education activities in schools. Primary health units are also privileged spaces for food and nutrition education activities, and it is essential to include persons with hypertension, since they do not seem to be adhering to a healthier diet. Special attention should be paid to fishermen and, in addition to educational activities, the support for access to minimum income policies is essential.
